# Timing Strategies of Direct-Acting Antivirals and Biologics Administration in HCV-Infected Subjects with Inflammatory Bowel Diseases

**DOI:** 10.3389/fphar.2017.00867

**Published:** 2017-11-21

**Authors:** Nicola Imperatore, Fabiana Castiglione, Antonio Rispo, Anna Sessa, Nicola Caporaso, Filomena Morisco

**Affiliations:** Gastroenterology, Department of Clinical Medicine and Surgery, School of Medicine “Federico II” of Naples, Naples, Italy

**Keywords:** inflammatory bowel diseases, hepatitis C virus infection, direct-acting antivirals, biologics, drug-to-drug interaction

## Abstract

**Background:** In the last years, inflammatory bowel disease (IBD) and hepatitis C virus (HCV) infection management has completely changed. However, the role of direct-acting antivirals (DAAs) and the correct timing of antiviral drugs administration in IBD patients needing biologics has not been evaluated.

**Objective:** To discuss the management of HCV-infected IBD patients, focusing our attention on the timing of DAAs administration subjects needing biologics.

**Methods:** Relevant articles addressing HCV management in patients needing biologics were identified by searching from PubMed, MEDLINE and Scopus.

**Results:** Three possible timing strategies were identified: (1) sequential strategy, meaning the choice of treating firstly the active IBD with biologics and then, once the acute phase has been controlled, treating the HCV infection; (2) concomitant strategy, that is the contemporaneous beginning of DAAs and biologics administration; (3) inverted sequential strategy—the administration of antiviral therapy before biologics in HCV-infected IBD patients. The potential pharmacological interactions between biologics and DAAs have also been reported.

**Conclusions:** Clinical management of HCV-infected IBD patients remains a challenging problem for clinicians, especially in terms of timing choice. Recent published data about DAAs are very encouraging also in IBD patients. All strategies could be considered safe and effective. However, further data are immediately required in order to evaluate hepatic toxicity of novel immunosuppressive drugs in IBD.

## Introduction

Inflammatory Bowel Diseases (IBD) and hepatitis C virus (HCV) infection are common conditions throughout the world, with an estimated prevalence of HCV infection in IBD subjects ranging from 1 to 6% in Western countries (Biancone et al., 2001; Loras et al., 2009; Chevaux et al., 2010). In the last years, IBD and HCV management has changed according to the introduction of different types of drugs [immunosuppressant (azathioprine, methotrexate) and biological therapies (infliximab, adalimumab, golimumab, vedolizumab) for IBD and direct-acting antivirals (DAAs) for HCV], with a great impact on the clinical course of these diseases (Scherzer et al., 2008; Gisbert et al., 2011; Allen et al., 2013; Caso et al., 2015; Safroneeva et al., 2015).

Since their introduction, anti-TNF agents have become widely used in moderate-to-severe IBD. Infliximab, adalimumab, and golimumab have been approved for use in induction and maintenance of remission in ulcerative colitis (UC), while only infliximab and adalimumab have been approved in Crohn's disease (CD). In some countries, also certolizumab has been approved in CD. Different mechanisms of action potentially contribute to the effectiveness of biologics, including neutralization of circulating TNF, inhibition of TNF binding to its receptor, and reverse signaling (Ben-Horin et al., 2016). Recently, another biologic drug has been approved for induction and maintenance of both CD and UC: vedolizumab. Vedolizumab specifically inhibits α4β7, impairing lymphocyte trafficking to the gut. It has been associated to lower risk of serious infections or neoplasia than anti-TNF alpha agents (Lobatón et al., 2014).

Recently, new classes of DAAs were introduced for HCV infection: the NS5A inhibitors that block the stage of membranous genesis (also known as –asvirs: daclatasvir, ledipasvir, ombitasvir, elbasvir, velpatasvir); the NS5B polymerase inhibitors (or –buvirs: as sofosbuvir and dasabuvir); the NS3/NS4A protease inhibitors (simeprevir and paritaprevir), which are characterized by a theoretically high potency, have a low barrier to development of resistance (selection of resistant viruses), and there is cross-resistance (drug-drug interaction) among the different NS3/NS4A protease inhibitors (Jakobsen et al., 2017).

However some questions need to be further clarified; in particular, data about the use of DAAs in IBD patients are still absent and the correct timing of antiviral drugs administration in IBD patients needing biologics has not been evaluated.

In this paper, we aimed to briefly discuss the possible timing strategies of DAAs administration in respect to biologic drugs in IBD patients infected with HCV.

## Methods

Clinical studies addressing HCV management in patients needing biologics were identified by searching from PubMed, MEDLINE and Scopus. The authors reviewed the studies and identified those which involved HCV subjects needing biologics or had implications for the management of IBD and HCV. Furthermore, several studies focused on drug-to-drug interaction were identified and reviewed to address the safety of biological drugs and DAAs administration.

## Sequential strategy

The choice of treating firstly the active IBD with biologics and then, once the acute phase of intestinal disease has been controlled, treating the HCV infection, appears to be one of the best timing strategies in this setting.

TNF-α may play a role in the pathogenesis of HCV and the inhibition of TNF-α may be potentially helpful for subsequent HCV clearance (Fukuda et al., 1996). In effect, modulation of TNF-α pathways has been claimed even beneficial in HCV patients, as TNF-α is involved in liver inflammation and hepatocyte apoptosis, and up-regulation of TNF pathways was thought to affect non-response to IFN-based treatments (Loras et al., 2010; Dill et al., 2011; Rahier et al., 2014).

Currently, there are no data to suggest that anti-TNF-α (infliximab, adalimumab, and golimumab) and vedolizumab are dangerous in IBD patients with HCV infection.

In the studies by Papa et al. (2013) and Morisco et al. (2013), none of the IBD patients experienced HCV reactivation during anti-TNF-α therapy, confirming the long-term safety of this therapy. The same results were obtained in a case series of 24 rheumatoid arthritis patients infected with HCV and treated with TNF-α antagonists (21 patients with etanercept, 3 patients with infliximab) (Peterson et al., 2003). Further observations indicate that anti-TNF-α can be safely administered in patients with different immune-mediated diseases and concomitant HCV infection (Viganò et al., 2012; Pompili et al., 2013; Sansone et al., 2014; Bonifati et al., 2016). The largest patient series providing information about long-term outcome of liver disease in IBD patients comes from a Spanish multicenter study (REPENTINA study) (Loras et al., 2014), including 74 HCV-infected patients with IBD and a follow-up of more than 20 years. No association was reported between cirrhosis development and prolonged or combined immunosuppressive or biologic treatment, suggesting that progression of liver disease in IBD patients seems to be similar to that reported in the non-immunosuppressed HCV population and that immunosuppressants administration does not result in accelerated liver disease progression (Coban et al., 2014), as reported instead in the organ transplantation setting.

However, no published clinical trial data were identified through literature search on the safety and efficacy of DAAs for the treatment of HCV infection in patients with IBD. On the other hand, although no drug interaction studies have been performed between DAAs and biologic agents for IBD (infliximab, adalimumab, golimumab, vedolizumab), it is reasonable that DAAs could be safe also in patients with IBD undergoing biologics (Koff, 2014). In fact clinically significant interactions would not be expected (Table 1), as biologics are not substrates of CYP450 and membrane transporters (Degasperi et al., 2016), while most of DAAs are inhibitors or metabolized by these molecules. However a recent study speculated a potential influence of biologics in the metabolism of small-molecule drugs indirectly through the CYP450, but these data need to be confirmed (Gupta et al., 2014).

**Table 1:** Drug-to-drug interactions between HCV DAAs and drugs used in IBD (Yeh et al., 2015; Degasperi et al., 2016; McConachie et al., 2016; Binda et al., 2017; Geddawy et al., 2017; González-Colominas et al., 2017; Kondili et al., 2017; Langness et al., 2017).

		**Immunosuppressors**		**Biologics**			
	**Steroids**	**AZA**	**MTX**	**IFX**	**ADA**	**GOL**	**VDZ**
SOF	No interaction expected	No interaction expected	No interaction expected	No interaction expected	No interaction expected	No interaction expected	No interaction expected
SOF/LDV	No interaction expected	No interaction expected	No interaction expected	No interaction expected	No interaction expected	No interaction expected	No interaction expected
SOF/VEL	No interaction expected	No interaction expected	No interaction expected	No interaction expected	No interaction expected	No interaction expected	No interaction expected
3D	Prednisolone exposure may be increased due to ritonavir CYP3A4 inhibition	No interaction expected	No interaction expected	No interaction expected	No interaction expected	No interaction expected	No interaction expected
GZR/EBR	No interaction expected	No interaction expected	MTX concentrations may be increased due to BCRP and P-gp inhibition	No interaction expected	No interaction expected	No interaction expected	No interaction expected
DCV	No interaction expected	No interaction expected	Potential for increased MTX concentrations due to DCV inhibition of OATP1B1	No interaction expected	No interaction expected	No interaction expected	No interaction expected
SIM	Potential for increased prednisolone concentrations due to SIM intestinal CYP3A inhibition	No interaction expected	No interaction expected	No interaction expected	No interaction expected	No interaction expected	No interaction expected

*SOF, sofosbuvir; SOF/LDV, sofosbuvir plus ledipasvir; SOF/VEL, sofosbuvir plus velpatasvir; 3D, ritonavir-boosted paritaprevir, plus ombitasvir and dasabuvir; GZR/EBR, grazoprevir plus elbasvir; DCV, daclatasvir; SIM, simeprevir; AZA, azathioprine; MTX; methotrexate; IFX, infliximab; ADA, adalimumab; GOL, golimumab; VDZ, vedolizumab; P-gp, P-glycoprotein; OATP1B1, organic anion-transporting polypeptide 1B1; BCRP, breast cancer resistance protein*.

For example, Simeprevir (an NS4/4A protease inhibitor) is metabolized by CYP3A, and is a mild inhibitor of intestinal CYP3A and CYP1A2. Daclatasvir (an NS5A inhibitor) is a substrate of CYP3A4 and of P-glycoprotein (P-gp); furthermore it is an inhibitor of organic anion-transporting polypeptide 1B1 (OATP1B1) of P-gp and of breast cancer resistance protein (BCRP). Paritaprevir is metabolized by CYP3A and is formulated with low dose ritonavir, a potent CYP3A inhibitor, to optimize paritaprevir exposure. Differently, neither sofosbuvir nor ledipasvir are metabolized by CYP450 enzymes and showed a less problematic profile of drug interaction.

About this topic, the European Association for the Study of the Liver (EASL) guidelines suggest that only the etanercept (an anti-TNF agent not used in IBD setting because of inefficacy) has a potential interaction with grazoprevir/elbasvir, which may require a dosage adjustment (European Association for the Study of the Liver, 2017).

Very recently, an expert panel summarized the advantages of sequential therapy in controlling extrahepatic manifestations of HCV infected subjects, underlying the ability of this timing strategy in:—differentiating the potential side effects related to the use of different drugs;—being characterized by a better tolerability in comparison to the concomitant ones (Ramos-Casals et al., 2017).

Therefore, the choice of treating firstly the IBD with biologics and then the HCV infection using antiviral therapy, could be one of the timing approaches, especially in patients rapidly needing biologics for an acute IBD, because of safety of biological drugs in HCV infected subjects and high rate of SVR after introducing antiviral therapy in patients treated with anti-TNF agents (Gupta et al., 2014; Koff, 2014; Degasperi et al., 2016; Ohta et al., 2016; European Association for the Study of the Liver, 2017; Ramos-Casals et al., 2017).

## Concomitant strategy

A second possible timing strategy for treating HCV-infected IBD patients is the contemporaneous beginning of DAAs and biologics administration.

Also in this case, it is essential to accurately evaluate the drug-to-drug interaction, as it can delay, decrease, or enhance absorption and metabolism of the drugs favoring or causing adverse effects.

Very recently, an expert panel summarized the advantages of concomitant therapy (Ramos-Casals et al., 2017), underlying the rapid complete response obtained with the use at the same time of Rituximab and DAAs in absence of safety issues and without interferences each others. This therapeutic strategy was associated with an excellent reduction of inflammatory/autoimmune response triggered by the virus following the administration of biologics and with the possibility, at the same time, to eliminate the circulatory virus as a consequence of DAAs administration. This last approach is used in situation, such as the hemophagocytic syndrome, in which IBD therapy is given along with antiviral therapy (Ramos-Casals et al., 2017). However, potential risks of enhanced toxicity (i.e., hematological) associated with their concomitant use have been reported (Zignego et al., 2017). In this case the priority should be given to immunosuppression especially in severe or life threatening diseases.

The available data about the concomitant therapy derived from studies carried out in patients suffering from cryoglobulinaemic vasculitis (Zignego et al., 2017). In two controlled clinical trials the combination of Rituximab (RTX) with antiviral therapy showed a synergistic effect, increasing the relapse-free survival.

Recently, Persico et al. (2017) questioned whether or not—and how—to treat the HCV positive patients suffering from diffuse large B cell lymphomas (DLBCL). The Authors treated patients with sofosbuvir plus ledipasvir for HCV 1b and, simultaneously, with chemotherapy (R-CHOP) for DLBCL, without relevant side effects. This interesting report suggests indirectly the efficacy of concomitant strategy also in case of IBD subjects, where anti-TNF and vedolizumab are obviously less problematic drugs than rituximab and/or chemotherapy.

Therefore, the choice of treating IBD and HCV infection at the same time could be considered a valid timing strategy in this setting, because of safety (no drug-drug interaction between biologics and antiviral drugs would be expected) and efficacy (reduction of inflammatory/autoimmune response triggered by the virus with the concomitant administration of biological agents), although specific data in IBD patients are still lacking. It is important to know that, due to the potential risk of hepatitis reactivation during immunosuppressing therapy, current management of IBD patients requires screening for HCV markers before starting immunosuppressive treatment, in order to determine the patient's virological status and consequently the need for treating chronic hepatitis. Monitoring liver tests every 3 months (ALT, ALP, GGT, bilirubin, albumin, platelets) is also recommended. In case of concomitant HCV infection, also HCV-RNA should be evaluated periodically (Degasperi et al., 2016; European Association for the Study of the Liver, 2017).

## Inverted sequential strategy

Finally, another timing option should be mentioned: the inverted sequential strategy, that is the administration of antiviral therapy before biologics in HCV-infected IBD patients.

Recent evidences have shown that HCV-RNA undetectability could be very early (within 4 weeks) by using DAAs (Sise et al., 2016; Tabernilla et al., 2017). Consequently, we can speculate that the administration of DAAs may be prior to introduction of biologics in HCV-infected IBD subjects. To the best of our knowledge, data about this timing strategy are still absent. In fact, before the administration of biologics in IBD patients, a screening is required: this could be the exact time point when starting DAAs administration. In the recent study by Saadoun et al. (2017), the regimen based on sofosbuvir plus daclatasvir seems to be able to confer an immunological advantage. Specifically, 41 patients with cryoglobulinaemic vasculitis were treated with sofosbuvir plus daclatasvir, showing that this DAAs regimen was able to revert Treg deficiency and to increase the expansion of IgM^+^CD21^−/low^ memory B cells, T follicular helper (TFH) cells and Th17 cells (Saadoun et al., 2017). Such results appear conceptually favorable to obtain a better response with a successive anti-TNF-α regimen. In a case series by Hahn et al. (2015), 3 patients completed treatment with DAAs and then received biologic or chemotherapeutic agents, without viral relapse at least 48 weeks after achievement of DAAs therapy. The persistence of SVR, despite immunosuppressive therapy, suggested that immune factors did not play a role in maintaining SVR after treatment with DAAs.

Therefore, considering these molecular mechanisms, it could be possible to obtain an advantage in administrating DAAs before biologics in IBD patients.

Sometimes, a cytolytic flare during biologic therapy is not be attributable to HCV, but to a drug-induced liver injury (DILI). A recent study by Björnsson et al. (2015), found 1 case out of 120 of DILI due to infliximab (0.8%) and 1 case out of 270 (0.3%) due to adalimumab. Although rare, this aspect needs to be evaluated during administration of DAAs and biologics.

Furthermore, kidney toxicity or unwanted autoimmune responses can complicate the situation (Oikonomou et al., 2011; Piga et al., 2014). As a consequence, all these aspects need to be evaluated by clinicians managing this kind of delicate patients.

## Conclusions

Clinical management of HCV-infected IBD patients remains a challenging problem for clinicians, especially in terms of timing choice. Recent published data about DAAs are very encouraging also in patients with IBD, allowing us to better manage HCV infection in the setting of IBD. About this topic, we believe that the timing strategy for treating HCV-infected IBD subjects could depend by several factors, including the IBD activity and patient's comorbidity. This means that a case-by-case decision could be the best choice, according to the most recent European Guidelines (Oikonomou et al., 2011). Sequential, concomitant and inverted sequential strategies could be all considered safe and effective. However, further data are immediately required in order to evaluate the possible long term poor liver disease outcome in HCV-infected IBD subjects and the hepatic toxicity of novel immunosuppressive drugs in IBD, such JAK inhibitors, anti-IL12/23 monoclonal antibodies and SMAD7 antisense inhibitors, which could potentially interfere with immunological surveillance of hepatotropic viruses.

## Author contributions

NI, FC, AR, and FM: planning the study, drafting the article, and revision of the manuscript. AS: drafting the article. NC: critical revision of the manuscript. All authors approved the final version of the article, including the authorship list.

### Conflict of interest statement

The authors declare that the research was conducted in the absence of any commercial or financial relationships that could be construed as a potential conflict of interest.

## References

[B1] Allen A. M. Kim W. R. Larson J. Loftus E. V. (2013). Efficacy and safety of treatment of hepatitis C in patients with inflammatory bowel disease. Clin. Gastroenterol. Hepatol. 11, 1655-1660.e1. 10.1016/j.cgh.2013.07.014 23891915 PMC3846435

[B2] Ben-Horin S. Vande Casteele N. Schreiber S. Lakatos P. L. (2016). Biosimilars in inflammatory bowel disease: facts and fears of extrapolation. Clin. Gastroenterol. Hepatol. 14, 1685–1696. 10.1016/j.cgh.2016.05.023 27215364

[B3] Biancone L. Pavia M. Del Vecchio Blanco G. D'Incà R. Castiglione F. De Nigris F.. (2001). Hepatitis B and C virus infection in Crohn's disease. Inflamm. Bowel Dis. 7, 287–294. 10.1097/00054725-200111000-00002 11720317

[B4] Binda C. Tortora A. Garcovich M. Annicchiarico B. E. Siciliano M. (2017). Toxicity and risks from drug-to-drug interactions of new antivirals for chronic hepatitis C. Eur. Rev. Med. Pharmacol. Sci. 21(1 Suppl.), 102–111. 28379589

[B5] Björnsson E. S. Gunnarsson B. I. Gröndal G. Jonasson J. G. Einarsdottir R. Ludviksson B. R.. (2015). Risk of drug-induced liver injury from tumor necrosis factor antagonists. Clin. Gastroenterol. Hepatol. 13, 602–608. 10.1016/j.cgh.2014.07.062 25131534

[B6] Bonifati C. Lora V. Graceffa D. Nosotti L. (2016). Management of psoriasis patients with hepatitis B or hepatitis C virus infection. World J. Gastroenterol. 22, 6444–6455. 10.3748/wjg.v22.i28.6444 27605880 PMC5006156

[B7] Caso F. Cantarini L. Morisco F. Del Puente A. Ramonda R. Fiocco U.. (2015). Current evidence in the field of the management with TNF-α inhibitors in psoriatic arthritis and concomitant hepatitis C virus infection. Expert Opin. Biol. Ther. 15, 641–650. 10.1517/14712598.2015.1011616 25652590

[B8] Chevaux J. B. Nani A. Oussalah A. Venard V. Bensenane M. Belle A.. (2010). Prevalence of hepatitis B and C and risk factors for nonvaccination in inflammatory bowel disease patients in Northeast France. Inflamm. Bowel Dis. 16, 916–924. 10.1002/ibd.21147 19885908

[B9] Coban S. Kekilli M. Köklü S. (2014). Approach and management of patients with chronic hepatitis B and C during the course of inflammatory bowel disease. Inflamm. Bowel Dis. 20, 2142–2150. 10.1097/MIB.0000000000000126 25072501

[B10] Degasperi E. Caprioli F. El Sherif O. Back D. Colombo M. Aghemo A. (2016). Challenges in treating patients with inflammatory bowel disease and concurrent viral hepatitis infection. Expert Rev. Gastroenterol. Hepatol. 10, 1373–1383. 10.1080/17474124.2016.1246181 27718758

[B11] Dill M. T. Duong F. H. Vogt J. E. Bibert S. Bochud P. Y. Terracciano L.. (2011). Interferon-induced gene expression is a stronger predictor of treatment response than IL28B genotype in patients with hepatitis C. Gastroenterology 140, 1021–1031. 10.1053/j.gastro.2010.11.039 21111740

[B12] European Association for the Study of the Liver (2017). EASL Recommendations on Treatment of Hepatitis C 2016. J. Hepatol. 66, 153–194. 10.1016/j.jhep.2016.09.001 36464532

[B13] Fukuda R. Ishimura N. Ishihara S. Chowdhury A. Morlyama N. Nogami C.. (1996). Intrahepatic expression of pro-inflammatory cytokine mRNAs and interferon efficacy in chronic hepatitis C. Liver 16, 390–399. 10.1111/j.1600-0676.1996.tb00768.x 9021719

[B14] Geddawy A. Ibrahim Y. F. Elbahie N. M. Ibrahim M. A. (2017). Direct Acting anti-hepatitis C virus drugs: clinical pharmacology and future direction. J. Transl. Int. Med. 5, 8–17. 10.1515/jtim-2017-0007 28680834 PMC5490957

[B15] Gisbert J. P. Chaparro M. Esteve M. (2011). Review article: prevention and management of hepatitis B and C infection in patients with inflammatory bowel disease. Aliment. Pharmacol. Ther. 33, 619–633. 10.1111/j.1365-2036.2010.04570.x 21416659

[B16] González-Colominas E. Londoño M. C. Morillas R. M. Torras X. Mojal S. Lens S.. (2017). Potential drug-drug interactions of Ombitasvir, Paritaprevir/Ritonavir ± Dasabuvir ± Ribavirin in clinical practice. J. Gastroenterol. Hepatol. [Epub ahead of print]. 10.1111/jgh.14014 28994141

[B17] Gupta R. Levin E. Wu J. J. Koo J. Liao W. (2014). An update on drug-drug interactions with biologics for the treatment of moderate-to-severe psoriasis. J. Dermatolog. Treat. 25, 87–89. 10.3109/09546634.2013.825041 23855593

[B18] Hahn K. J. Kohli A. Sims Z. Kottilil S. (2015). Durable sustained virologic response after oral directly acting antiviral therapy despite immunosuppressive treatment. Open Forum Infect. Dis. 2:ofv091. 10.1093/ofid/ofv091 26634218 PMC4665358

[B19] Jakobsen J. C. Nielsen E. E. Feinberg J. Katakam K. K. Fobian K. Hauser G.. (2017). Direct-acting antivirals for chronic hepatitis C. Cochrane Database Syst. Rev. 9:CD012143. 10.1002/14651858.CD012143.pub3 28922704 PMC6484376

[B20] Koff R. S. (2014). Review article: the efficacy and safety of sofosbuvir, a novel, oral nucleotide NS5B polymerase inhibitor, in the treatment of chronic hepatitis C virus infection. Aliment. Pharmacol. Ther. 39, 478–487. 10.1111/apt.12601 24387618

[B21] Kondili L. A. Gaeta G. B. Ieluzzi D. Zignego A. L. Monti M. Gori A.. (2017). Real-life data on potential drug-drug interactions in patients with chronic hepatitis C viral infection undergoing antiviral therapy with interferon-free DAAs in the PITER Cohort Study. PLoS ONE 12:e0172159. 10.1371/journal.pone.0172159 28245248 PMC5330484

[B22] Langness J. A. Nguyen M. Wieland A. Everson G. T. Kiser J. J. (2017). Optimizing hepatitis C virus treatment through pharmacist interventions: Identification and management of drug-drug interactions. World J. Gastroenterol. 23, 1618–1626. 10.3748/wjg.v23.i9.1618 28321163 PMC5340814

[B23] Lobatón T. Vermeire S. Van Assche G. Rutgeerts P. (2014). Review article: anti- adhesion therapies for inflammatory bowel disease. Aliment. Pharmacol. Ther. 39, 579–594. 10.1111/apt.12639 24479980

[B24] Loras C. Gisbert J. P. Mínguez M. Merino O. Bujanda L. Saro C.. (2010). Liver dysfunction related to hepatitis B and C in patients with inflammatory bowel disease treated with immunosuppressive therapy. Gut 59, 1340–1346. 10.1136/gut.2010.208413 20577000

[B25] Loras C. Gisbert J. P. Saro M. C. Piqueras M. Sánchez-Montes C. Barrio J.. (2014). Impact of surveillance of hepatitis b and hepatitis c in patients with inflammatory bowel disease under anti-TNF therapies: multicenter prospective observational study (REPENTINA 3). J. Crohns. Colitis. 8, 1529–1538. 10.1016/j.crohns.2014.06.009 25052345

[B26] Loras C. Saro C. Gonzalez-Huix F. Mínguez M. Merino O. Gisbert J. P.. (2009). Prevalence and factors related to hepatitis B and C in inflammatory bowel disease patients in Spain: a nationwide, multicenter study. Am. J. Gastroenterol. 104, 57–63. 10.1038/ajg.2008.4 19098850

[B27] McConachie S. M. Wilhelm S. M. Kale-Pradhan P. B. (2016). New direct-acting antivirals in hepatitis C therapy: a review of sofosbuvir, ledipasvir, daclatasvir, simeprevir, paritaprevir, ombitasvir and dasabuvir. Expert Rev. Clin. Pharmacol. 9, 287–302. 10.1586/17512433.2016.1129272 26651915

[B28] Morisco F. Castiglione F. Rispo A. Stroffolini T. Sansone S. Vitale R.. (2013). Effect of immunosuppressive therapy on patients with inflammatory bowel diseases and hepatitis B or C virus infection. J. Viral Hepat. 20, 200–208. 10.1111/j.1365-2893.2012.01643.x 23383659

[B29] Ohta Y. Kanda T. Katsuno T. Yasui S. Haga Y. Sasaki R.. (2016). Successful sofosbuvir treatment with ribavirin dose reduction for chronic hepatitis C virus genotype 2 infection in a patient with ulcerative colitis: a case report. BMC Gastroenterol. 16:66. 10.1186/s12876-016-0480-x 27401874 PMC4940930

[B30] Oikonomou K. Kapsoritakis A. Eleftheriadis T. Stefanidis I. Potamianos S. (2011). Renal manifestations and complications of inflammatory bowel disease. Inflamm. Bowel Dis. 17, 1034–1045. 10.1002/ibd.21468 20842645

[B31] Papa A. Felice C. Marzo M. Andrisani G. Armuzzi A. Covino M.. (2013). Prevalence and natural history of hepatitis B and C infections in a large population of IBD patients treated with anti-tumor necrosis factor-α agents. J. Crohns. Colitis. 7, 113–119. 10.1016/j.crohns.2012.03.001 22464811

[B32] Persico M. Aglitti A. Caruso R. De Renzo A. Selleri C. Califano C.. (2017). Efficacy and safety of new direct antiviral agents in HCV infected patients with Diffuse Large B Cell Non-Hodgkin Lymphoma. Hepatology. [Epub ahead of print]. 10.1002/hep.29364 28714143

[B33] Peterson J. R. Hsu F. C. Simkin P. A. Wener M. H. (2003). Effect of tumour necrosis factor alpha antagonists on serum transaminases and viraemia in patients with rheumatoid arthritis and chronic hepatitis C infection. Ann. Rheum. Dis. 62:1078. 10.1136/ard.62.11.1078 14583571 PMC1754346

[B34] Piga M. Chessa E. Ibba V. Mura V. Floris A. Cauli A.. (2014). Biologics-induced autoimmune renal disorders in chronic inflammatory rheumatic diseases: systematic literature review and analysis of a monocentric cohort. Autoimmun. Rev. 13, 873–879. 10.1016/j.autrev.2014.05.005 24840285

[B35] Pompili M. Biolato M. Miele L. Grieco A. (2013). Tumor necrosis factor-α inhibitors and chronic hepatitis C: a comprehensive literature review. World J. Gastroenterol. 19, 7867–7873. 10.3748/wjg.v19.i44.7867 24307780 PMC3848134

[B36] Rahier J. F. Magro F. Abreu C. Armuzzi A. Ben-Horin S. Chowers Y.. (2014). Second European evidence-based consensus on the prevention, diagnosis and management of opportunistic infections in inflammatory bowel disease. J. Crohns. Colitis. 8, 443–468. 10.1016/j.crohns.2013.12.013 24613021

[B37] Ramos-Casals M. Zignego A. L. Ferri C. Brito-Zerón P. Retamozo S. Casato M.. (2017). Evidence-based recommendations on the management of extrahepatic manifestations of chronic hepatitis C virus infection. J. Hepatol. 66, 1282–1299. 10.1016/j.jhep.2017.02.010 28219772

[B38] Saadoun D. Pol S. Ferfar Y. Alric L. Hezode C. Si Ahmed S. N.. (2017). Efficacy and safety of sofosbuvir plus daclatasvir for the treatment of HCV-associated cryoglobulinemic vasculitis. Gastroenterology 153, 49-52.e5 10.1053/j.gastro.2017.03.006 28288791

[B39] Safroneeva E. Vavricka S. R. Fournier N. Pittet V. Peyrin-Biroulet L. Straumann A.. (2015). Impact of the early use of immunomodulators or TNF antagonists on bowel damage and surgery in Crohn's disease. Aliment. Pharmacol. Ther. 42, 977–989. 10.1111/apt.13363 26271358

[B40] Sansone S. Guarino M. Castiglione F. Rispo A. Auriemma F. Loperto I.. (2014). Hepatitis B and C virus reactivation in immunosuppressed patients with inflammatory bowel disease. World J. Gastroenterol. 20, 3516–3524. 10.3748/wjg.v20.i13.3516 24707134 PMC3974518

[B41] Scherzer T. M. Staufer K. Novacek G. Steindl-Munda P. Schumacher S. Hofer H.. (2008). Efficacy and safety of antiviral therapy in patients with Crohn's disease and chronic hepatitis C. Aliment. Pharmacol. Ther. 28, 742–748. 10.1111/j.1365-2036.2008.03779.x 19145730

[B42] Sise M. E. Bloom A. K. Wisocky J. Lin M. V. Gustafson J. L. Lundquist A. L.. (2016). Treatment of hepatitis C virus-associated mixed cryoglobulinemia with direct-acting antiviral agents. Hepatology 63, 408–417. 10.1002/hep.28297 26474537 PMC4718772

[B43] Tabernilla A. Grandal M. Pernas B. Castro-Iglesias A. Rodríguez-Osorio I. Mena A.. (2017). Viral dynamics among hepatitis C virus chronic infected patients during direct-acting antiviral agents therapy: impact for monitoring and optimizing treatment duration. Eur. J. Gastroenterol. Hepatol. 29, 781–785. 10.1097/MEG.0000000000000882 28410351

[B44] Viganò M. Degasperi E. Aghemo A. Lampertico P. Colombo M. (2012). Anti-TNF drugs in patients with hepatitis B or C virus infection: safety and clinical management. Expert Opin. Biol. Ther. 12, 193–207. 10.1517/14712598.2012.646986 22188392

[B45] Yeh W. W. Feng H. P. Dunnington K. M. Marricco C. N. Caro L. Guo Z. (2015). No clinically meaningful pharmacokinetic interaction between HCV inhibitors grazoprevir/elbasvir with tacrolimus, mycophenolate mofetil, and prednisolone, but cyclosporine increases grazoprevir/elbasvir exposures in healthy subjects, in 66th Annual Meeting of the American Association for the Study of Liver Diseases (San Francisco, CA).

[B46] Zignego A. L. Ramos-Casals M. Ferri C. Saadoun D. Arcaini L. Roccatello D.. (2017). International therapeutic guidelines for patients with HCV-related extrahepatic disorders. A multidisciplinary expert statement. Autoimmun. Rev. 16, 523–541. 10.1016/j.autrev.2017.03.004 28286108

